# A Rapid Thermal Absorption Rate and High Latent Heat Enthalpy Phase Change Fiber Derived from Bio-Based Low Melting Point Copolyesters

**DOI:** 10.3390/polym14163298

**Published:** 2022-08-12

**Authors:** Tsung-Yu Lan, Hsu-I Mao, Chin-Wen Chen, Yi-Ting Lee, Zhi-Yu Yang, Jian-Liang Luo, Pin-Rong Li, Syang-Peng Rwei

**Affiliations:** 1 Department of Molecular Science and Engineering, Institute of Organic and Polymeric Materials, Research and Development Center of Smart Textile Technology, National Taipei University of Technology, No. 1, Sec. 3, Chung-Hsiao East Road, Taipei 106, Taiwan; 2 Department of Orthopedic Surgery, Far Eastern Memorial Hospital, No. 21, Sec. 2, Nanya S. Rd., New Taipei City 220, Taiwan; 3 Department of Materials and Textiles, Asia Eastern University of Science and Technology, No. 58, Sec. 2, Sichuan Rd., New Taipei City 220, Taiwan

**Keywords:** poly(butylene adipate-co-hexamethylene adipate), 1,4-cyclohexanedimethanol, phase change material, melt spinning

## Abstract

A series of poly(butylene adipate-*co*-hexamethylene adipate) (PBHA) copolymers with different content of 1,4-cyclohexanedimethanol (CHDM) was synthesized via one-step melt polymerization. The PBHA copolymer with 5 mol% CHDM (PBHA-C5) exhibited a low melting point (T_m_) and high enthalpy of fusion (∆H_m_) of 35.7 °C and 43.9 J g^−1^, respectively, making it a potential candidate for an ambient temperature adjustment textile phase change material (PCM). Polybutylene terephthalate (PBT) was selected as the matrix and blended at different weight ratios of PBHA-C5, and the blended samples showed comparable T_m_ and ∆H_m_ after three cycles of cooling and reheating, indicating good maintenance of their phase changing ability. Samples were then processed via melt spinning with a take-up speed of 200 m min^−1^ at draw ratios (DR) of 1.0 to 3.0 at 50 °C. The fiber’s mechanical strength could be enhanced to 2.35 g den^−1^ by increasing the DR and lowering the PBHA-C5 content. Infrared thermography showed that a significant difference of more than 5 °C between PBT and other samples was achieved within 1 min of heating, indicating the ability of PBHA-C5 to adjust the temperature. After heating for 30 min, the temperatures of neat PBT, blended samples with 27, 30, and 33 wt% PBHA-C5, and neat PBHA-C5 were 53.8, 50.2, 48.3, 47.2, and 46.5 °C, respectively, and reached an equilibrium state, confirming the temperature adjustment ability of PBHA-C5 and suggesting that it can be utilized in thermoregulating applications.

## 1. Introduction

A phase change material (PCM) is a material that can absorb or release heat during a phase transition within a specific temperature range. There is high potential for the application of PCMs in many fields [1,2,3,4,5,6,7], and the study of PCMs has been extensively discussed in the literature [8,9,10,11,12,13,14,15]. PCMs can be divided into three types according to their components: organic, inorganic, and hybrid PCMs [16,17]. Organic PCMs exhibit various advantages, such as low price, high energy storage density, and a wide range of phase change temperatures [18,19,20,21,22,23,24]. However, the issue of melt leakage during phase transition and the low thermal conductivity of pure organic PCMs limit their application [25,26]. Therefore, suitable supporting materials play an important role in preventing leakage in PCMs. The strategy in processing can be divided into three categories: microencapsulated PCMs (MPCMs) [3], solid–solid PCMs (SSPCMs) [27], and form-stable PCMs (FSPCMs) [28]. For MPCMs, a high melting point polymer acts as a shell in the outer layer with the PCM inside, avoiding leakage during phase transition. The other two methods are based on the interaction between supporting materials and PCMs, mainly controlled by a chemical mechanism in SSPCMs and a physical mechanism in FSPCMs.

The research on PCM-incorporated thermoregulating fabrics has attracted considerable attention due to the ability to tune their properties by controlling the structure and composition of the fibers. Moreover, fabrics’ high ratio of surface area with porous structure allows for various functional application possibilities through modification of the fabric [29,30,31,32,33,34,35,36,37,38]. Several spinning methods have been applied to produce PCM filaments [1]. Melt spinning is one of the most efficient techniques that is solvent-free and can mix supporting materials and PCMs with a screw extruder machine at a specific temperature. Iqbal and co-workers produced PCM fibers via a melt spinning process with the addition of MPCMs to a polypropylene monofilament. The melting enthalpy of the fiber was determined to be 9.2 J g^−1^ with MPCM content of 12 wt%. A linear model was developed to predict the relationship between fiber properties and PCM loading content [39,40]. Fredi’s research group introduced paraffin-based MPCMs into a PP filament for preparing PCM fibers. The take-up speed tuned the diameter of the fibers, and the mechanical properties and morphology were affected by the content of MPCM [41]. Tomaszewski’s team blended PP with paraffin via a melt spinning process, observing increasing melting filament enthalpy with increased content of paraffin [42]. Xia and colleagues prepared FSPCMs by adding polyethylene glycol derivatives as PCMs and the esters into fumed silica (F-SiO_2_ NPs); then, the FSPCMs were introduced into polyamide 6 fibers for melt spinning. The phase change fibers showed not only high melting enthalpy of 11.1 J g^−1^ but also outstanding washing durability [43,44]. Cherif and co-workers developed a stable and reproducible bi-component melt spinning process for incorporating PCM into fibers. The as-spun fiber with PCM exhibited high heat capacities ranging from 21 to 23 J g^−1^. At a draw ratio of 2.0, the physical properties of the fibers were comparable to acetate fibers, with sufficient strength and elongation to withstand further processing in various textile processes [45].

We aimed to produce thermoregulating textiles with a phase-changing temperature near the skin temperature of the human body surface. Liang et al. synthesized poly(butylene adipate-co-hexamethylene adipate) (PBHA) copolymers with different ratios of butylene adipate to hexamethylene adipate (BA/HA). The melting temperature and enthalpy were 32 °C and 85 J g^−1^, respectively, at a molar ratio of BA/HA = 55/45, presenting potential for development as a PCM due to the appropriate melting point and high heat of fusion [46,47]. Therefore, we chose PBHA with a ratio of BA/HA = 55/45 as the PCM matrix and copolymerized it with various amounts of 1,4-cyclohexanedimethanol (CHDM) to enhance the melting viscosity to avoid leakage while in use [48,49]. The synthesized copolymers (PBHA-C) were blended with commercial polybutylene terephthalate (PBT) chips as a supporting material and then processed via the melt spinning technique. The effects of the PCM content and draw ratio on fibers’ thermal and mechanical properties were investigated. 

## 2. Experimental Section

### 2.1. Materials

Firstly, 1,4-butanediol (BDO, 99%) was obtained from J.T. Baker, and 1,6-hexanediol (HDO, 99%) and polybutylene terephthalate (PBT, I.V. = 1.2 dL g^−1^) masterbatch were purchased from Emperor Chemical Co., Ltd. Adipic acid (AA, 99.8%) was provided by Asahi Kasei Corporation. Easchem Co., Ltd. produced and supplied 1,4-cyclohexanedimethanol (CHDM, 99%). Titanium(IV)butoxide (Ti(OBu)_4_, 97%), tetrahydrofuran (THF, 99.9%), and chloroform-d (d-CDCl_3_, 99.8%) were supplied by Sigma-Aldrich.

### 2.2. Synthesis of PBHA-Cn Copolymers

**Scheme 1:**
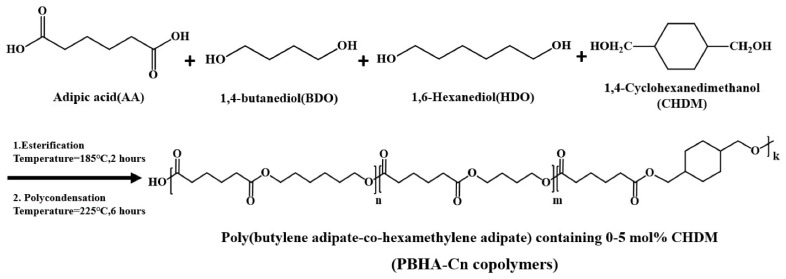
Synthetic route of PBHA-Cn copolymers.

PBHA-Cn copolymers were synthesized via one-step melt polymerization, and the process is summarized in Scheme 1. First, AA, BDO, HDO, CHDM, and the catalyst were placed in an autoclave under a nitrogen atmosphere and heated to 185 °C until the amount of the water reached 90% of its theoretical amount to obtain the prepolymer. Next, the system was heated to 225 °C gradually under 1 kPa pressure and held at this temperature for 1 h. Then, the temperature was maintained at 225 °C under a high vacuum below 1 torr. While the torque value (in watt) was raised to 1.3 times from the initial reference value, the melted copolymers were quenched rapidly in ice water for further analysis. The synthesized copolymers are named PBHA-Cn, where n = the mole ratio of CHDM.

### 2.3. Blending

PBHA-C5 copolymers and PBT were blended using a twin-screw extruder (Process 11, Thermo Fisher, Waltham, MA, USA). PBHA-C5 copolymer was added in a melting state at 100 °C with melted feeding equipment, while the PBT masterbatch was simultaneously added with pellet feeding equipment. The content of PBHA-C5 copolymer in the blended product was controlled to be 27, 30, and 33 wt%. Samples were named according to the weight ratio of PBHA-C5/PBT.

### 2.4. Melt Spinning

Melt spinning of the blended product was conducted on a twin-screw extruder (Process 11, Thermo Fisher, Waltham, MA, USA) with a length-to-diameter (L/D) ratio of 10 and equipped with a melting gear pump. System temperature, screw speed, and melting gear pump speed were set at 235 ± 5 °C, 80 rpm, and 4 rpm, respectively. The sample was extruded through a 10-hole spinneret with a hole diameter of 0.2 mm, and the multifilament was taken up at a speed of 200 m min^−1^ at room temperature.

### 2.5. Drawing

The as-spun fibers were drawn in a chamber under heating at 50 °C. The draw ratios were set at 1.0, 1.5, 2.0, 2.5, and 3.0 for all samples.

### 2.6. Instrumental Methods

The chemical structures of the copolymers were analyzed by nuclear magnetic resonance, ^1^H NMR (JEOL ECZ600R 600 MHz, Tokyo, Japan), at room temperature, with chloroform-d as the solvent. 

Functional group analysis was carried out using an FT-IR spectrometer (PerkinElmer, Waltham, MA, USA) using 32 co-added scans in a range of 400–4000 cm^−1^. 

The molecular weight and polydispersity index (PDI) were analyzed by GPC (Viscotek GPCmax VE-2001, Malvern Panalytical, Malvern, England). DMF solvent (5 mL) was mixed with 5% LiBr and 20 mg of sample to dissolve for examination. Polystyrene standards were applied for calibration.

Thermogravimetric analysis, TGA (Hitachi, STA 7200, Tokyo, Japan), was used to determine the decomposition point of 5% weight loss (T_d-5%_) for all samples at a temperature range of 30 to 550 °C and a heating rate of 10 °C min^−1^ under a nitrogen atmosphere.

Dynamic mechanical tests were carried out using a dynamic mechanical analyzer, DMA (Tech Max DMS 6100, Tokyo, Japan), in the temperature range of −80 to 30 °C, with a heating rate of 5 °C min^−1^ and a frequency of 1 Hz in the tension mode. 

The thermal properties were determined by differential scanning calorimetry (DSC) (Hitachi High Tech. DSC-7000, Tokyo, Japan). The samples were encapsulated in aluminum pans and were examined from −20 to 250 °C at heating and cooling rates of 10 °C min^−1^ under a nitrogen atmosphere. 

Wide-angle X-ray scattering diffraction (WAXD) patterns were evaluated using a Malvern Panalytical X’Pert3 powder diffractometer (Malvern, Worcestershire, UK) in 2θ from 10 to 40 degrees at room temperature, with a scanning speed of 0.2° min^−1^ with Cu K_α_ radiation (λ = 0.154 nm).

DSC (Hitachi High Tech. DSC-7000, Tokyo, Japan) was also employed to evaluate the nonisothermal crystallization behavior of the copolymers. The samples were kept under a nitrogen atmosphere in aluminum pans. Samples were first heated from −20 to 80 °C at 10 °C min^−1^ and then maintained at 80 °C for 5 min to remove the thermal history. Next, the samples were cooled to −20 °C at different rates of 5, 10, 15, and 20 °C min^−1^. Finally, the heat flow traces were recorded for analysis.

The crystallization morphology of copolymers was observed using a Polarized Optical Microscope (POM, Nikon ECLIPSE LV100N POL) equipped with a heating stage and a liquid nitrogen cooling control unit (Linkam THMS Examina/FTIR600, Salfords, UK). The samples were heated to 80 °C, held at this temperature for 5 min to erase the thermal history, and then cooled to a specific temperature at a rapid cooling rate of 100 °C min^−1^ for crystallization. A Nikon camera recorded the images.

Rheological properties were determined using a capillary rheometer (Rheo-Tester 1000, Göttfert, Germany) (L/D = 10) under shear rates from 1000 to 7000 s^−1^ at 220, 230, and 240 °C to check the melting spinning ability. The apparent viscosity in Pa·s was measured at different temperatures and shear rates.

The stability of the phase-changing ability of the blended samples after multiple temperature cycles was evaluated by DSC (Hitachi High Tech. DSC-7000, Tokyo, Japan). Samples were sealed in aluminum pans, heated from −20 to 80 °C at 10 °C min^−1^, held at 80 °C for 5 min, and then cooled to −20 °C. The process was repeated three times for each sample. 

Tensile testing of fiber samples was carried out with a fiber stretching test machine (34SC-1, Instron, Norwood, Massachusetts, USA) under a crosshead speed of 50 mm min^−1^, and stress values at the break and elongation were collected from the stress–strain curve.

Infrared thermography was performed using a thermal imager (Ti95, Fluke, Washington, DC, USA). PBT and blended samples were prepared as fabric, and the PBHA-C5 was shaped as a film. The samples were placed in an oven and heated to 55 °C, and the surface temperature variation was recorded over a 30 min period. 

## 3. Results and Discussion

### 3.1. Copolymer Synthesis and Characterization

**Figure 1:**
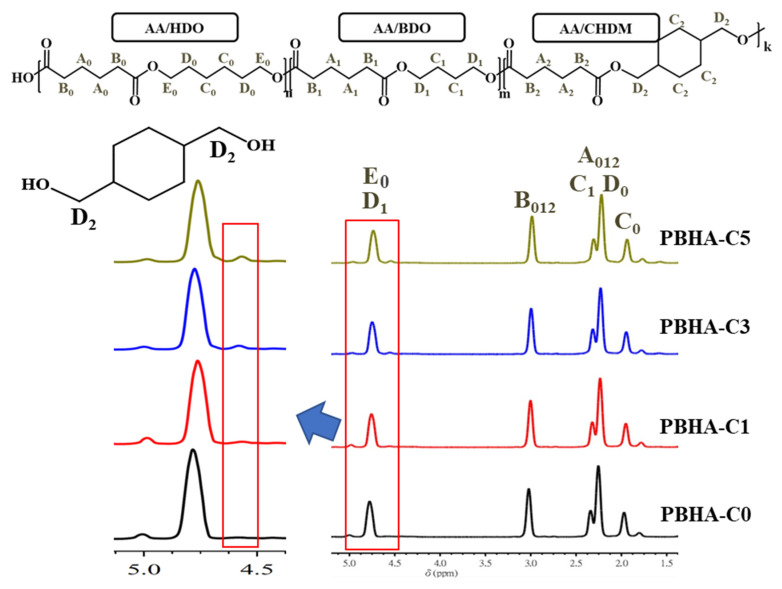
^1^H-NMR spectrum of PBHA-Cn copolymers.

^1^H NMR spectra confirmed the chemical structure in PHHA-Cn copolymers, and the chemical shift information of the characteristic signals was marked, as shown in Figure 1. The characteristic signals E_0_ and D_1_ at around δ = 4.7 ppm are attributed to CH_2_ on the α position of HDO and BDO. The chemical shift B_012_ at δ = 3.0 ppm belongs to the CH_2_ signals on the adipic acid. Signal C_0_ at δ = 1.9 ppm corresponds to the CH_2_ on the γ position of HDO. Signal D_2_ at δ = 4.6 ppm appeared, and its strength increased as the content of CHDM increased, which is ascribed to the CH_2_ signals on the α position of CHDM. By calculating the integral areas of the signals, the chemical structures of PBHA-Cn copolymers were confirmed using the following formulas:

(1) $$X_{\mathrm{HDO}}=\frac{I_{1.9}/4}{I_{\mathrm{3.0}}/4}$$

(2) $$X_{\mathrm{CHDM}}=\frac{I_{4.6}/4}{I_{\mathrm{3.0}}/4}$$

(3) $$X_{\mathrm{BDO}}=\frac{1-X_{\mathrm{HDO}}-X_{\mathrm{CHDM}}}{1}$$

The error range of comonomer unit composition was estimated to be ±2% [46,50].

Additionally, the molecular weight of copolymers was determined by GPC. The data revealed that all copolymers had similar number average molecular weight (M_n_) values above 30,000 g mol^−1^, demonstrating that complete copolymerization was achieved. All data are tabulated in Table 1.

**Figure 2:**
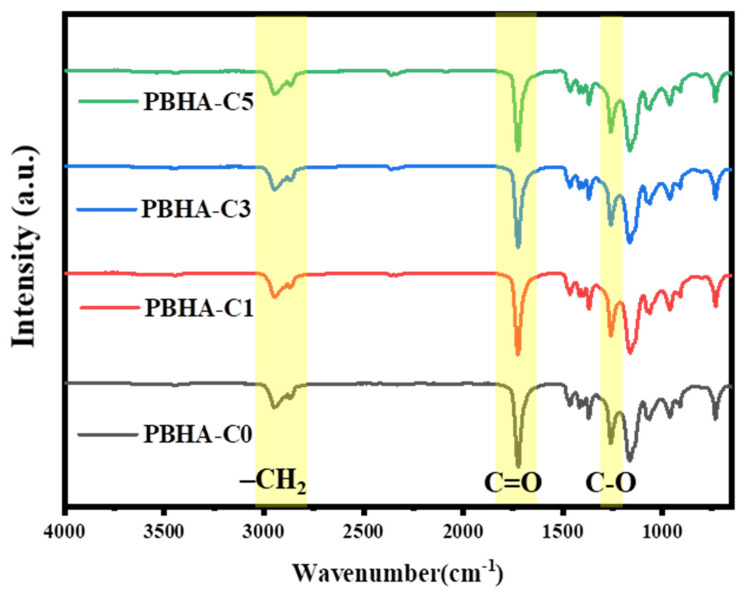
FT-IR spectra of PBHA-Cn copolymers.

Figure 2 displays the FT-IR spectra of synthesized PBHA-Cn copolymers. The absorption peaks of –CH_2_ vibration appeared at 2940 cm^−1^ and 2866 cm^−1^. The stretching vibration of the C-O absorption of the ester group appeared at 1254 cm^−1^, and the signal of the C=O of the ester bond appeared at 1725 cm^−1^, suggesting that the synthesis of copolymers was achieved [46,51].

**Figure 3:**
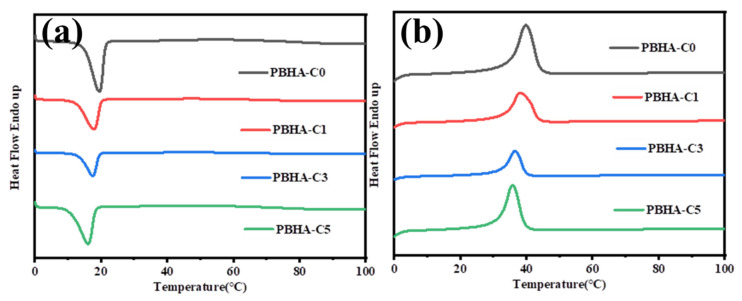
DSC curve of PBHA-Cn copolymers in (**a**) first cooling process and (**b**) second reheating process at the same rate of 10 °C min^−1^.

Figure 3 shows the heat flow traces of PBHA-Cn copolymers in a temperature range of 0 to 100 °C. As revealed in the first cooling process, the values of crystallization temperature (T_c_) and enthalpy (∆H_c_) decreased from 19.5 to 16.1 °C and from 49.5 to 45.2 J g^−1^, respectively, with increased content of CHDM. The reduction in crystallization ability is attributed to the disruption of the regularity of the molecular chains by the presence of CHDM. A similar tendency was observed in the second heating process, where T_m_ and ∆H_m_ decreased from 39.8 to 35.7 °C and from 48.9 to 43.9 J g^−^^1^, respectively. The introduction of CHDM with cyclohexane structure hinders the stacking of molecular chains and leads to more defects in the crystal, resulting in a decrement in the melting temperature [52]. All data are listed in Table 2.

**Figure 4:**
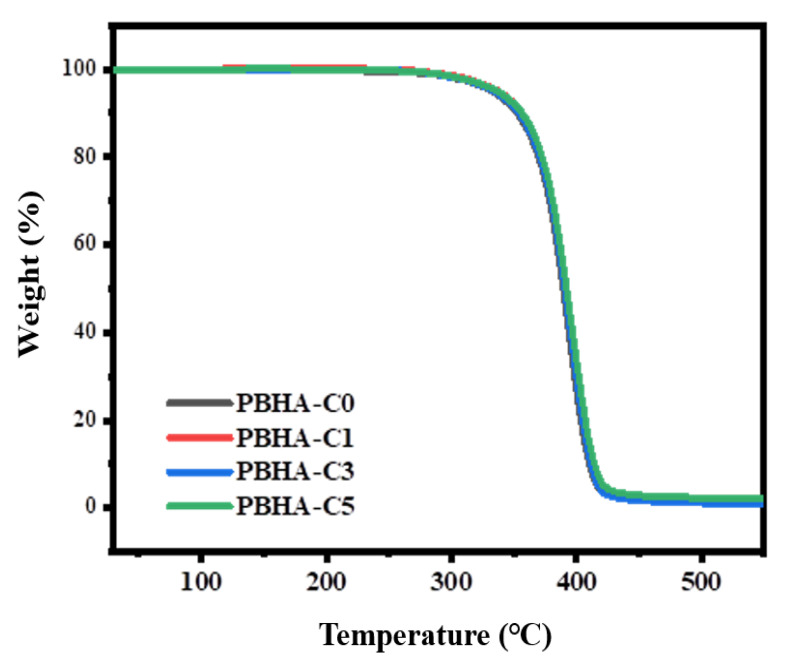
Weight loss as a function of temperature for PBHA-Cn copolymers.

The thermal decomposition behaviors were determined using TGA. Figure 4 shows the curves of weight residue as a function of temperature from 30 to 550 °C, and the 5% weight loss (T_d-5%_) for samples is listed in Table 2. No notable variation was observed with the introduction of CHDM, and all samples displayed high decomposition temperatures above 300 °C, reflecting that good thermal stability was maintained when CHDM was present.

**Figure 5:**
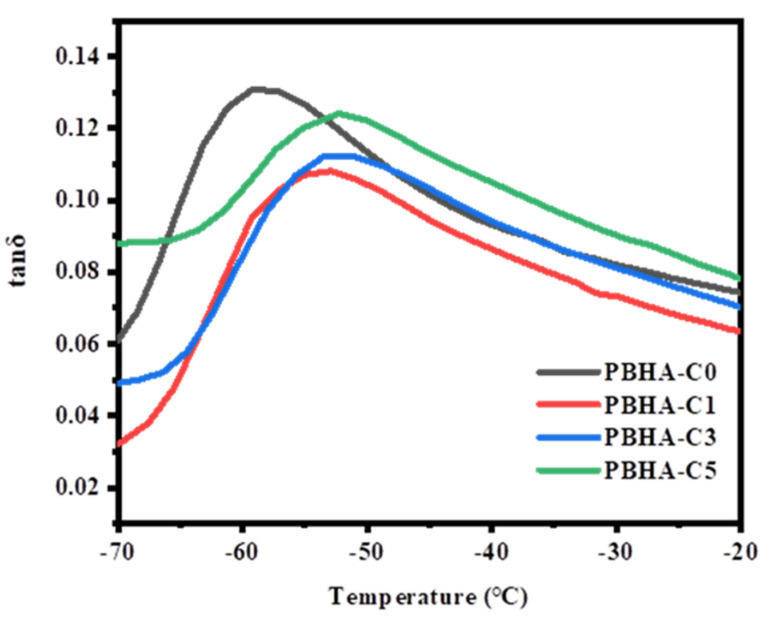
Tan δ of PBHA-Cn copolymers as a function of temperature.

Figure 5 shows the DMA curves of the PBHA-Cn samples. The glass transition temperature (T_g_) of neat PBHA was around −58.9 °C and gradually increased to −52.4 °C as the CHDM content increased to 5 mol%. The value of T_g_ can be controlled by the content of aliphatic linear chains and cyclohexane groups. As the content of CHDM increased, the concentration of cyclohexane groups increased, which contributed to limiting the molecular chain mobility. Therefore, a higher T_g_ value was observed [50,53]. The data are presented in Table 2.

**Figure 6:**
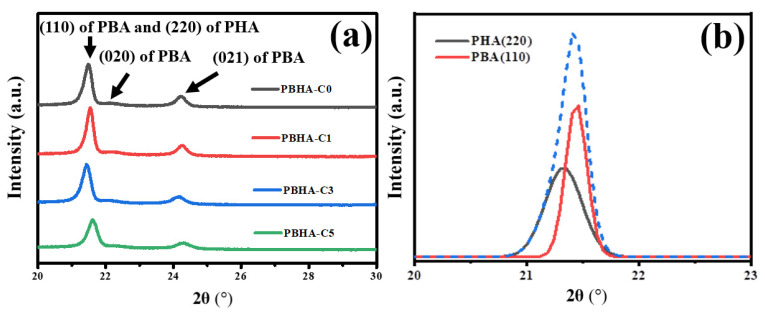
(**a**) XRD patterns of PBHA-Cn copolymers, and (**b**) the overlap feature peak by (110) of PBA and (220) of PHA.

WAXD analysis was employed to determine the crystalline structure of PBHA-Cn copolymers. Figure 6 presents the results of the PBHA-Cn samples in a 2θ range of 20–30°. The characteristic peaks around the 2θ values of 22.3° and 24.2° are related to the crystal lattices of (020) and (021) of poly(butylene adipate) (PBA), respectively. Moreover, a peak composed of overlapping signals by (110) of PBA and (220) of poly(hexamethylene adipate) (PHA) was observed [46]. The overlapped peak was analyzed further using Origin software. By entering the information of the theoretical signal locations of crystalline PBA and PHA, the overlapped signals were separated successfully, and the two bands ((110) of PBA and (220) of PHA) were obtained. This result demonstrates that there was no phase transition with the incorporation of CHDM, despite variations in the peak intensity.

**Figure 7:**
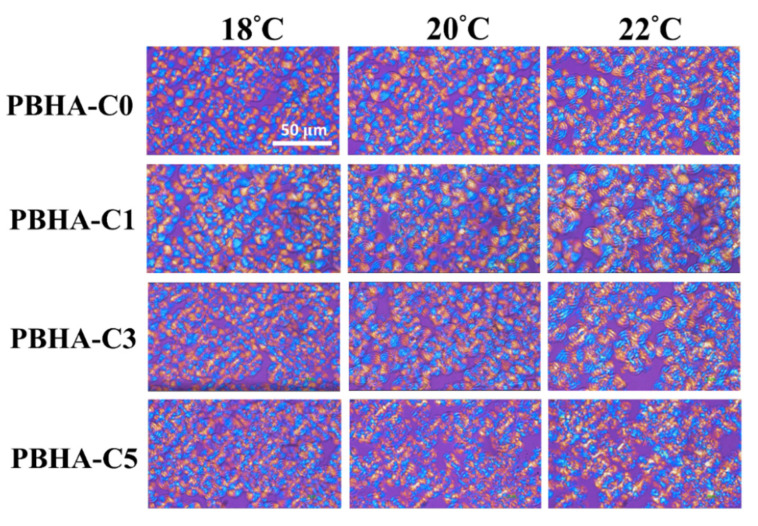
POM images of PBHA-Cn copolymers at a given temperature.

The POM images of PBHA-Cn copolymers are shown in Figure 7. The samples were cooled to specific temperatures and held at constant temperature for isothermal crystallization, while the formation of crystals was observed. For the samples with CHDM content of 0, 1, and 3 %, the formation of ring-banded spherulite was observed at all temperatures [54]. At a CHDM content of 5%, the additional cyclohexane units may have disturbed the crystalline structure during the growth process, and the presence of ring-banded spherulite became unclear, especially at a temperature ≥22 °C. Furthermore, the nonisothermal crystallization kinetic behavior of PBHA-Cn copolymers was analyzed using the Avrami model [55,56,57,58,59]. Avrami exponents were found to be in the range of 3.5 to 4.2, reflecting homogeneous nucleation and three-dimensional growth of the crystal structure. All detailed calculation methods are described in the supporting information. The nonisothermal DSC curves of PBHA-Cn copolymers in the temperature range from –20 to 80 °C under 5, 10, 15, and 20 °C min^−1^, relative crystallinity fraction, *X*(*t*), as a function of time, the curve of log{−ln[1−*X*(*t*)]} versus log t at *X*(*t*) in the range from 20 to 80%, and related data are presented in Figures S1–S3 and summarized in Table S1, respectively.

### 3.2. Blends of Copolymers and PBT

PBHA-C5 was chosen as our target PCM for further testing due to the suitable T_m_ of 35.7 °C and ∆H_m_ of 43.9 J g^−1^. PBHA-C5 was blended at different weight ratios with PBT pellets. The blended samples were named PBHA-C5/PBT = 33/67, PBHA-C5/PBT = 30/70, and PBHA-C5/PBT = 27/73 based on the weight ratios between PBHA-C5 and PBT. Thermal experiments on the blended samples were conducted by DSC, as shown in Figure S4. Two endothermic areas were observed at around 33 and 220 °C in the second heating curves in all blended samples, corresponding to the melting behaviors of PBHA-C5/PBT and PBT crystalline separately. Figure S5 displays the TGA results, revealing decomposition curves with T_d-5%_ above 300 °C for the samples and demonstrating that good thermal stability was sustained after blending.

**Figure 8:**
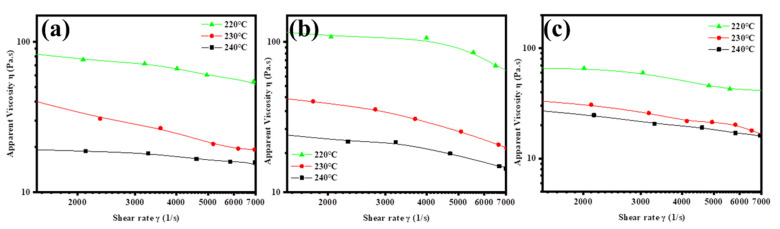
Apparent viscosity plots of blended samples for (**a**) PBHA-C5/PBT = 33/67, (**b**) PBHA-C5/PBT = 30/70, and (**c**) PBHA-C5/PBT = 27/73 under different temperatures and shear rates.

The apparent viscosities were measured to evaluate the spinning processability of the blended samples [60,61]. The viscosity curves at a shear rate range of 1000 to 7000 s^−1^ and at temperatures 220, 230, and 240 °C were generated using a capillary rheometer, as shown in Figure 8. Generally, the preferred viscosity values should be between 30 and 80 Pa·s at a shear rate range of 2000 to 7000 s^−1^ to ensure stability in the melt spinning process. According to the results, the spinning operation temperature was determined to be 235 °C for blended samples in the following melt spinning experiment.

**Figure 9:**
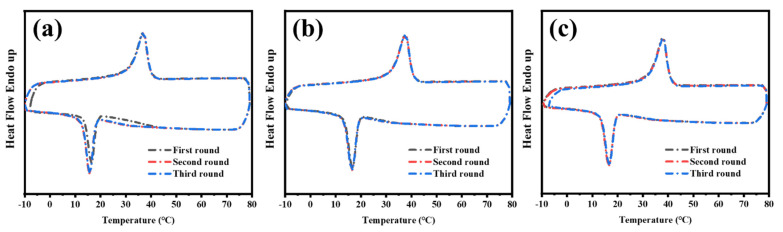
DSC curves of PBHA-Cn copolymers in three cycles of cooling and reheating: (**a**) PBHA-C5/PBT = 33/67, (**b**) PBHA-C5/PBT = 30/70, and (**c**) PBHA-C5/PBT = 27/73.

The maintenance of the phase-changing ability of the inserted PCM under multiple usage cycles plays an essential role in ambient temperature adjustment textile applications. Therefore, the samples were cooled and reheated from −20 to 80 °C at 10 °C min^−1^ using DSC analysis for three cycles, as shown in Figure 9. The results show that the samples exhibited consistent heat flow traces for all three blended samples, demonstrating that the phase-changing ability of the inserted PCM was retained successfully.

### 3.3. Fiber Production and Characterization of Properties

**Figure 10:**
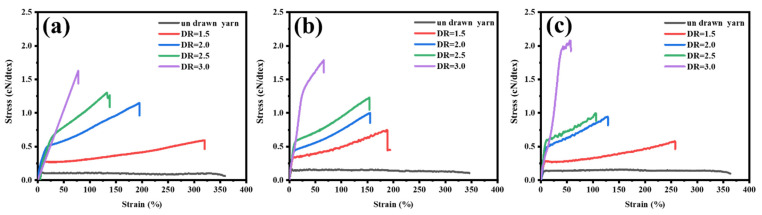
Tensile properties of fiber at various draw ratios: (**a**) PBHA-C5/PBT = 33/67, (**b**) PBHA-C5/PBT = 30/70, and (**c**) PBHA-C5/PBT = 27/73.

The production of a multifilament of blended samples was carried out via a melt spinning process. The maintenance of good thermal stabilities with T_d-5%_ above 300 °C for all fiber samples after the melt spinning process was first confirmed by TGA measurement, as shown in Figure S6. The as-spun fibers were then drawn at 50 °C at varying draw ratios of 1.5, 2.0, 2.5, and 3.0. The stress–strain curves of the fibers are presented in Figure 10, showing a conventional fiber extension behavior where the tensile strength increased and the elongation rate decreased with an increased draw ratio. The crystallinity enhancement was due to the further orientation of the molecular chain after the drawing process [62].

The maximum stresses and elongation values of the drawn fibers are listed in Table S2. Fiber samples with a draw ratio of 3.0 were selected for further testing due to their higher stress and suitable elongation at break values ~50%, which is appropriate for fabric applications. The maximum stress values for fibers with 27, 30, and 33 wt% PBHA-C5 were 2.07 ± 0.13, 1.78 ± 0.08, and 1.62 ± 0.02 g cN dtex^−1^, respectively. This observation suggested that the fiber’s mechanical strength decreased with the increase in PBHA-C5 and improved as the content of PBT increased [63].

**Figure 11:**
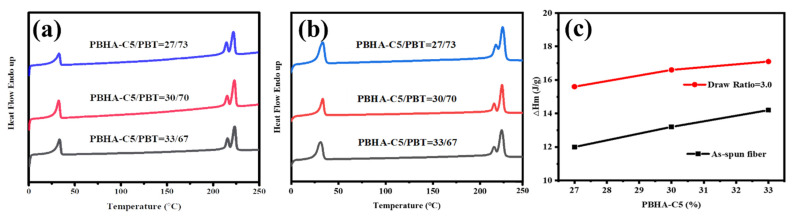
DSC curves of fiber samples in the first heating trace: (**a**) as-spun fiber, (**b**) fiber at draw ratio = 3.0, and (**c**) plots of melting enthalpy for the as-spun fiber and with draw ratio of 3.0 as a function of PBHA-C5 content.

The heat flow curves of as-spun fibers and samples with a 3.0 draw ratio in the first heating process were examined by DSC analysis, as displayed in Figure 11. All samples showed comparable values of T_m_ after drawing. Furthermore, an enhancement in ∆H_m_ was observed with an increased draw ratio, indicating an enhancement in crystallinity induced by molecular chain orientation due to the drawing process. The observation also agrees with the previous results of the tensile test.

**Figure 12:**
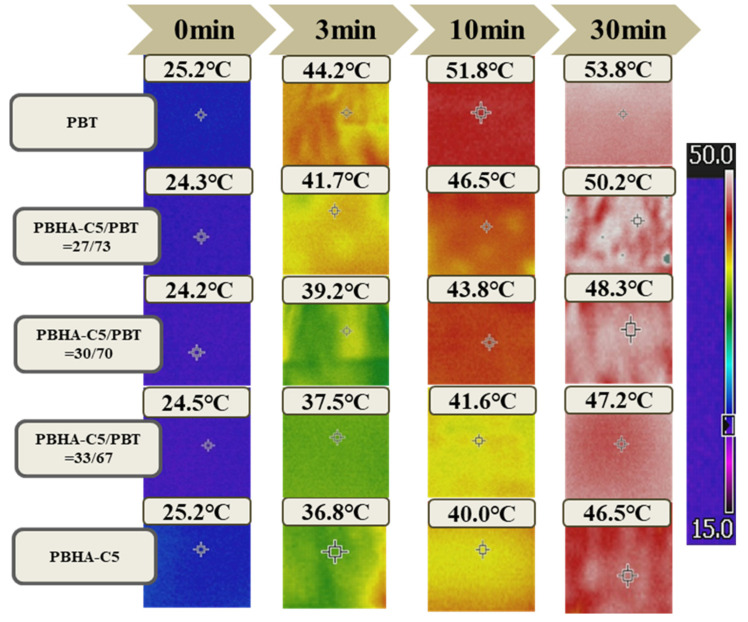
Infrared temperature image for neat PBT, blended fiber samples with 27, 30, 33 wt% PBHA-C5, and neat PBHA-C5 for different heating times at 55 °C.

**Figure 13:**
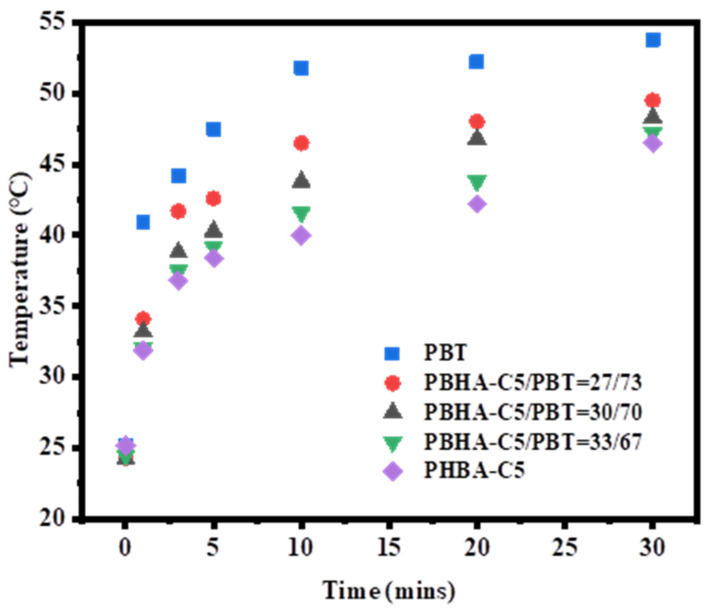
Plots of the surface temperature change in samples as a function of time.

The infrared thermography technique absorbed and stored heat during heating in materials by changing the temperature difference between the test and reference samples. Samples of neat PBT, blended samples with 27, 30, and 33 wt% PBHA-C5, and neat PBHA-C5 were placed in an oven, continuously heated from 25 °C to 55 °C, and the variation in temperature on the surfaces was measured. The infrared thermography images are presented in Figure 12, and the plots of surface temperature as a function of time are illustrated in Figure 13. A significant difference of more than 5 °C between PBT and the other samples was achieved within 1 min of heating, indicating the noticeable effect of PBHA-C5 on temperature adjustment. Over time, the surface temperature of the samples increased at different rates that were lower for samples with higher PBHA-C5 content. After 30 min, the temperature of neat PBT, blends with 27, 30, and 33 wt% PBHA-C5, and neat PBHA-C5 were 53.8, 50.2, 48.3, 47.2, and 46.5 °C, respectively [64], suggesting that PBHA-C5 as a PCM matrix has sufficient latent enthalpy for thermoregulating applications.

## 4. Conclusions

A series of PBHA copolymers with different CHDM content was copolymerized via one-step melt polymerization, and all samples exhibited excellent thermal stability at T_d-5%_ above 300 °C. DSC analysis showed a decrease in melting and crystallization enthalpies and temperatures with an increase in CHDM, suggesting that the introduction of cyclohexane in CHDM hinders the stacking of molecular chains and leads to more defects in the crystal. Similar morphologies of the negative spherulite with a ring-banded morphology in all samples were observed via POM. Nonisothermal crystallization kinetic analysis showed a range of Avrami exponents from 3.5 to 4.2 to display the spherulite morphology. The PBHA-C5 copolymer was chosen in the experiment due to its suitable values of T_m_ of 35.7 °C and ∆H_m_ of 43.9 J g^−1^ for use as the inserted PCM matrix in ambient temperature adjustment textiles. The infrared thermography technique absorbed and stored heat during heating in materials by changing the temperature difference between the test and reference samples. The surface temperature of neat PBT, blends with 27, 30, and 33 wt% PBHA-C5, and neat PBHA-C5 were 53.8, 50.2, 48.3, 47.2, and 46.5 °C, respectively, after heating to 55 °C over 30 min. The rate of increase in temperature was lower as the content of PBHA-C5 was increased, indicating that the temperature adjustment ability of fabric samples was achieved successfully and can be adopted in thermoregulating textile applications.

## Figures and Tables

**Table 1:** Molecular weight from GPC results and the feed and calculated ratios of PBHA-Cn copolymers from NMR.

Sample	M_n_	M_w_	M_w_/M_n_	BDO/HDO/CHDM Feed Ratio	BDO/HDO/CHDM Calculated
	(g mol^−1^)	(g mol^−1^)	(*Đ*)	(mole %)	(mole %)
PBHA-C0	34,800	106,100	3.07	52.5/47.5/0	52/48/0
PBHA-C1	36,600	113,300	3.09	52/47/1	51/48/1
PBHA-C3	36,800	109,500	2.96	51/46/3	50/47/3
PBHA-C5	31,100	83,300	2.57	50/45/5	49/47/4

**Table 2:** Thermal properties of PBHA-Cn copolymers.

Sample	T_m_ ^a^ (°C)	∆H_m_ ^a^ (J g^−1^)	T_c_ ^a^ (°C)	∆H_c_ ^a^ (J g^−1^)	T_d-5%_^b^ (°C)	T_g_^c^ (°C)
PBHA-C0	39.8	48.9	19.5	49.5	332.7	−58.9
PBHA-C1	38.1	49.0	17.8	48.7	337.5	−55.2
PBHA-C3	36.5	44.9	17.5	45.3	334.1	−53.9
PBHA-C5	35.7	43.9	16.1	45.2	336.5	−52.4

Note: a is analysis by DSC; b is analysis by TGA, c is analysis by DMA.

## Data Availability

The data that support the findings of this study are available from the corresponding authors upon reasonable request.

## Supplementary Materials

The following support information can be downloaded at: https://www.mdpi.com/article/10.3390/polym14163298/s1, Figure S1: DSC curves of PBHA-Cn copolymers in cooling process at 5, 10, 15, and 20 °C min^−1^: (a) PBHA-C0, (b) PBHA-C1, (c) PBHA-C3, and (d) PBHA-C5; Figure S2: Relative crystallinity (X(t)) as a function of crystallization time under various cooling rates: (a) PBHA-C0, (b) PBHA-C1, (c) PBHA-C3, and (d) PBHA-C5; Figure S3: Avrami plots for PBHA-Cn copolymers: (a) PBHA-C0, (b) PBHA-C1, (c) PBHA-C3, and (d) PBHA-C5; Figure S4: DSC curve of PBT and blended samples in (a) first cooling process and (b) second reheating process under the same rate of 10 °C min−1; Figure S5: Weight loss as a function of temperature for PBT and blended samplesl; Figure S6: Weight loss as a function of temperature for fiber samples; Table S1: Avrami analysis for nonisothermal crystallization and half-time of crystallization for PBHA-Cn copolymers; Table S2: Tensile properties of fiber samples.
