# Supplementary material for: A Rapid Thermal Absorption Rate and High Latent Heat Enthalpy Phase Change Fiber Derived from Bio-Based Low Melting Point Copolyesters
Source: Polymers (Basel). 2022 Aug 12;14(16):3298. doi: 10.3390/polym14163298 (PMC9413292; doi:10.3390/polym14163298)
Supplement: Supplementary file 1 [file polymers-14-03298-s001.zip › polymers-1852451-supplementary.pdf]

Supporting Information

A Rapid Thermal Absorption Rate and High Latent Heat Enthalpy Phase Change Fiber Derived from Bio-Based Low Melting Point Copolyesters

Tsung-Yu Lan[1,2,3], Hsu-I Mao[1], Chin-Wen Chen[1,*], Yi-Ting Lee[1], Zhi-Yu Yang[1], Jian-Liang Luo[1], Pin-Rong Li[1] and Syang-Peng Rwei[1,*]

[1] Department of Molecular Science and Engineering, Institute of Organic and Polymeric Materials, Research and Development Center of Smart Textile Technology, National Taipei University of Technology, No. 1, Sec. 3, Chung-Hsiao East Road., Taipei, 10608, Taiwan

[2] Department of Orthopedic Surgery, Far Eastern Memorial Hospital, No. 21, Sec. 2, Nanya S. Rd., New Taipei City 220, Taiwan

[3] Department of Materials and Textiles, Asia Eastern University of Science and Technology, No.58, Sec.2, Sichuan Rd., New Taipei City 220, Taiwan

##

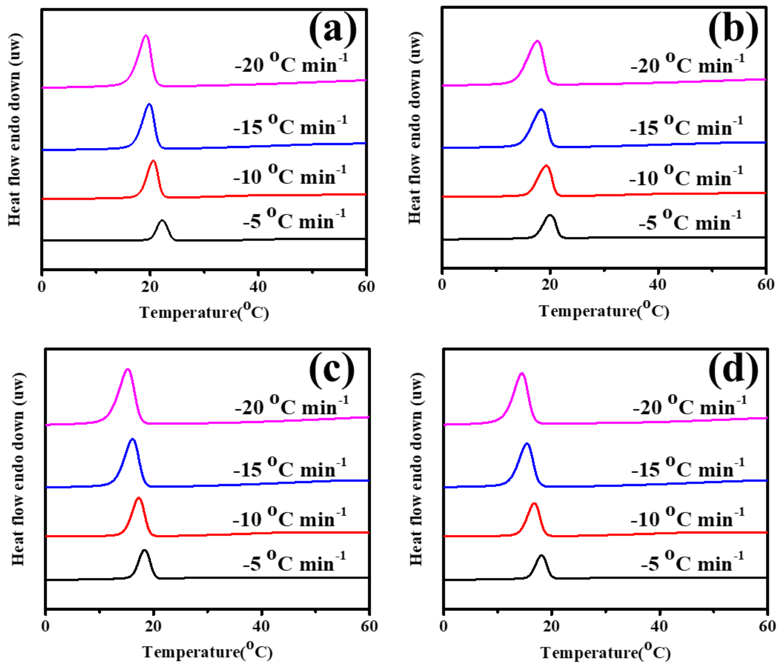

Figure S1. DSC curves of PBHA-Cn copolymers in cooling process at 5, 10, 15, and 20 °C min^{-1}: (a) PBHA-C0, (b) PBHA-C1, (c) PBHA-C3, and (d) PBHA-C5.

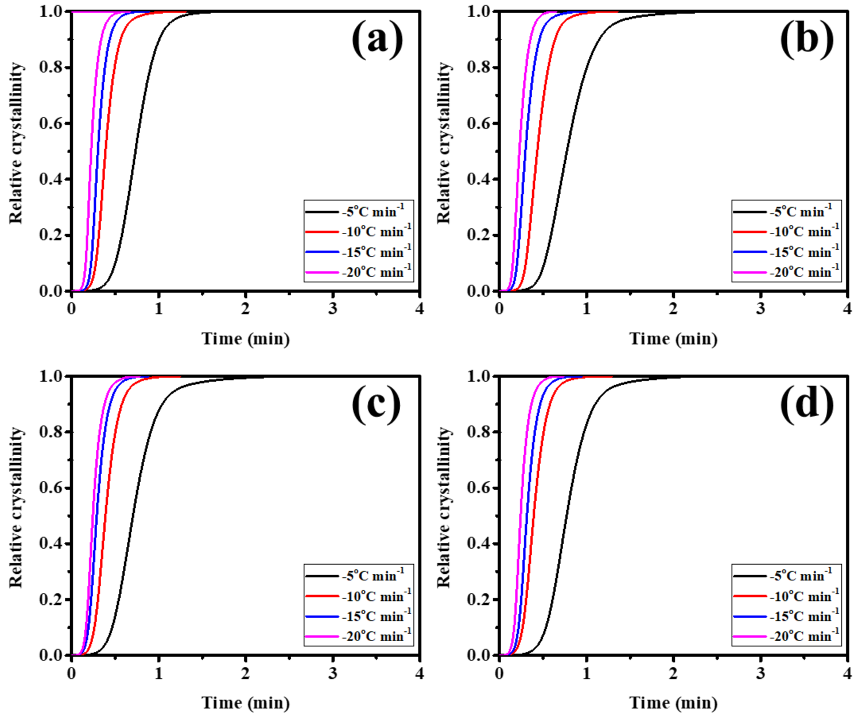

Figure S2. Relative crystallinity (X(*t*)) as a function of crystallization time under various cooling rates: (a) PBHA-C0, (b) PBHA-C1, (c) PBHA-C3, and (d) PBHA-C5.

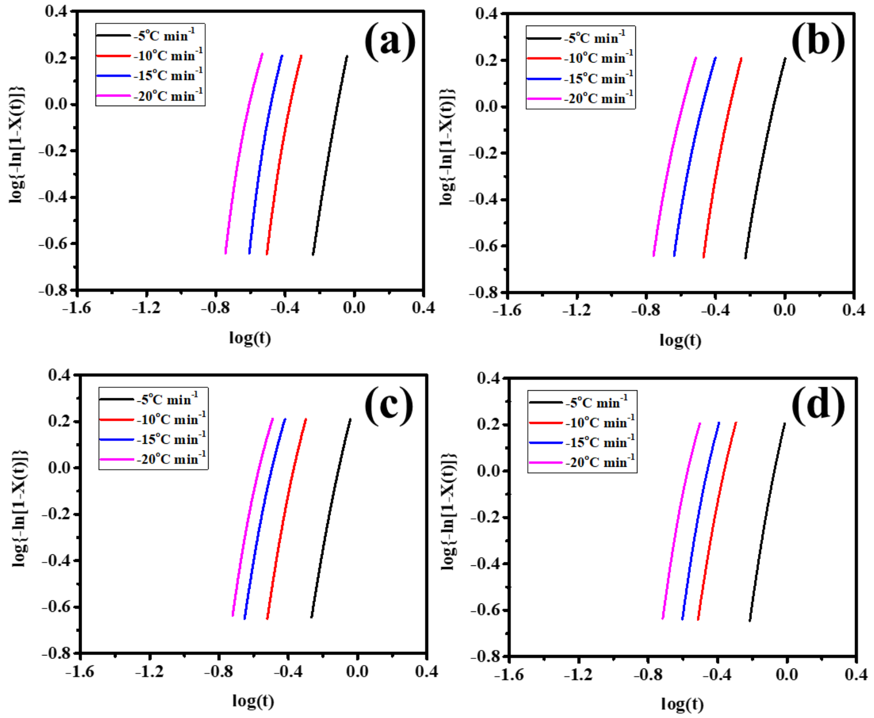

Figure S3. Avrami plots for PBHA-Cn copolymers: (a) PBHA-C0, (b) PBHA-C1, (c) PBHA-C3, and (d) PBHA-C5.

The nonisothermal DSC traces of copolymers in the cooling process under a rate of 5, 10, 15, and 20 min^{-1} from 80 to -20 °C are shown in Figure S1. The following equation can be used to calculate the relative crystallinity fraction X(t):

$$X(t) = \frac{X_C(t)}{X_C(t_\infty)} = \frac{\int_0^t \frac{\mathrm{d}H(t)}{\mathrm{d}t}\mathrm{d}t}{\int_0^\infty \frac{\mathrm{d}H(t)}{\mathrm{d}t}\mathrm{d}t} \quad \text{(S1)}$$

where dH(t) denotes the crystallization enthalpy at a specific temperature during the time interval dt via cooling, while demonstrating that dH(t)/dt is the rate of heat flow; the relative crystallinity fraction X(t) as a function of time was analyzed as shown in Figure S2.

The exothermic heat during the crystallization process can be further analyzed, and the behavior is determined using the Avrami model, as described in the following equation:

$$X(t) = 1 - \exp(-kt^n) \quad \text{(S2)}$$

$$\log\{-\ln[1 - x(t)]\} = -\log k + n \log t \quad \text{(S3)}$$

where n is the Avrami exponent, and k is the constant value of the crystallization rate.

The experimental data are substituted into the equations with an X(t) range of 20%-80%, and the traces of log t versus log{–ln[1–X(t)]} for copolymers are exhibited in Figure S3. The Avrami equation parameters, n, and k were obtained via the slopes and intercepts of the traces. The following equations were used to determine the half-time and growth rates:

$$t_{1/2} = (\ln 2/k)^{(1/n)} \quad \text{(S4)}$$

$$G = 1/t_{1/2} \quad \text{(S5)}$$

All the data are summarized in Table S1.

Table S1. Avrami analysis for nonisothermal crystallization and half-time of crystallization for PBHA-Cn copolymers.

	**Temp (Ø)**	**n**	**k (min^{-n})**	**$t_{1/2}$ (min)**	**G (min^{-1})**
**PBHA-C0**	5	4.2	2.624	0.74	1.358
	10	4.2	35.934	0.39	2.543
	15	4.1	129.330	0.31	3.269
	20	4.0	241.602	0.23	4.390
**PBHA-C1**	5	3.7	1.716	0.78	1.279
	10	3.8	16.181	0.44	2.278
	15	3.5	44.864	0.30	3.308
	20	3.5	99.312	0.23	4.318

**PBHA-C3**	5	3.8	2.523	0.71	1.405
	10	3.8	25.200	0.39	2.547
	15	3.7	62.158	0.29	3.417
	20	3.6	111.020	0.25	4.049
**PBHA-C5**	5	3.7	1.985	0.78	1.284
	10	3.8	24.570	0.40	2.516
	15	3.9	64.849	0.32	3.135
	20	3.8	163.682	0.25	4.068

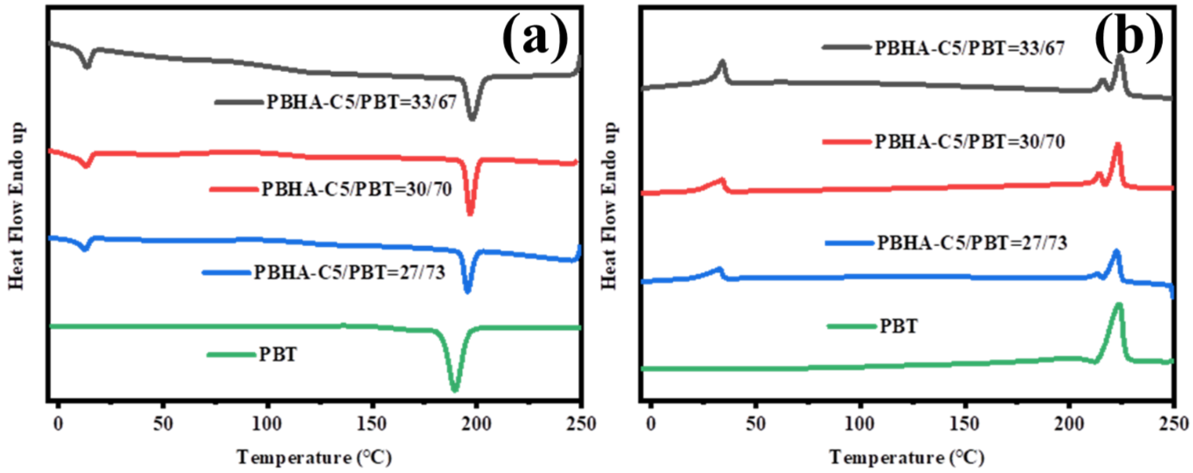

Figure S4. DSC curve of PBT and blended samples in (a) first cooling process and (b) second reheating process under the same rate of 10 °C min^{-1}.

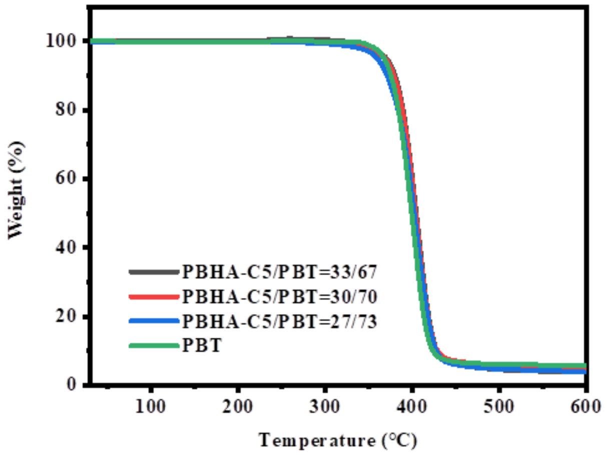

Figure S5. Weight loss as a function of temperature for PBT and blended samples.

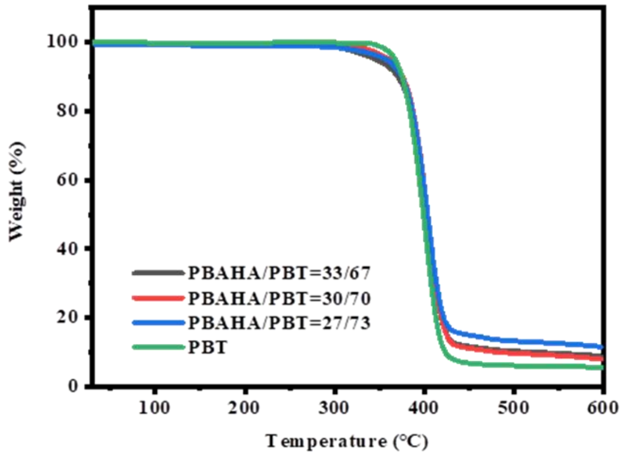

Figure S6. Weight loss as a function of temperature for fiber samples.

Table S2. Tensile properties of fiber samples.

**Sample**	**Draw ratio**	**Stress (cN dtex^{-1})**	**Elongation (%)**
**PBHA-C5/PBT=33/67**	0	0.06 ±0.01	356.77 ±15.12
	1.5	0.59 ±0.13	319.96 ±22.38
	2	1.13 ±0.08	194.96 ±4.11
	2.5	1.30 ±0.04	132.69 ±7.85
	3	1.62 ±0.02	77.16 ±2.46
**PBHA-C5/PBT=30/70**	0	0.11 ±0.01	345.77 ±12.9
	1.5	0.74 ±0.16	188.09 ±20.71
	2	0.99 ±0.11	155.12 ±10.56
	2.5	1.21 ±0.13	153.12 ±7.97
	3	1.78 ±0.07	65.85 ±2.98
**PBHA-C5/PBT=27/73**	0	0.10 ±0.01	363.63 ±14.20
	1.5	0.57 ±0.04	257.45 ±10.54
	2	0.94 ±0.10	128.17 ±16.75

2.5	1.00 ±0.12	104.94 ±5.32
3	2.07 ±0.13	68.78 ±1.45
